# A Key Node Mining Method Based on Acupoint-Disease Network (ADN): A New Perspective for Exploring Acupoint Specificity

**DOI:** 10.1155/2020/6031601

**Published:** 2020-07-12

**Authors:** Han Shi, Yang Liu, Enuo Cui, Hai Zhao, Wei Gao, Jian Zhu, Dongxiang Yang

**Affiliations:** ^1^Engineering Research Center of Security Technology of Complex Network System, School of Computer Science and Engineering, Northeastern University, Shenyang 110169, China; ^2^School of Information Science and Engineering, Shenyang University of Technology, Shenyang 110870, China; ^3^School of Information Science and Engineering, Shenyang University, Shenyang 110044, China; ^4^College of Computer Science and Technology, Shenyang University of Chemical Technology, Shenyang 110142, China; ^5^Affiliated Hospital of Liaoning University of Traditional Chinese Medicine (AH-LUTCM), Shenyang 110032, China

## Abstract

In the process of treating pro-diseases with acupuncture, traditional Chinese medicine (TCM) doctors may fine-tune acupuncture prescriptions according to different prior experiences. Different prescriptions will affect the efficiency and effect of acupuncture treatment, and even excessive acupoint selection may cause psychological pressure on patients. We still lack an effective means to analyze the meridian system and acupoint specificity to clarify the mapping relationship between acupoints and diseases. Given the inability of modern medical technology to provide effective evidence support for meridians and acupoints, we combined acupuncture theory with network science for an interdisciplinary discussion. In this paper, we constructed a weighted undirected acupoint-disease network (ADN) based on clinical acupuncture prescription literature and proposed a high-specificity key node mining method based on ADN. Combined with the principle of acupoint selection in TCM, the proposed method balanced the contribution of local areas to the network based on the distribution characteristics of meridians and selected 30 key acupoints with high influence on the global topology according to the evaluation index of key nodes. Finally, we compared the proposed method with the other six classical node importance evaluation algorithms in terms of resolution, network loss, and accuracy. The comprehensive results show that the marked key acupoint nodes make outstanding contributions to the connectivity, topological structure, and weighted benefits of the network, and the stability and specificity of the algorithm guarantee the reliability of the key acupoint nodes. We consider that these key acupoints with high centrality in ADN can be used as core acupoints to help researchers explore targeted and high-impact acupoint combinations under resource constraints and optimize existing acupuncture prescriptions.

## 1. Introduction

As a traditional medical method, acupuncture has been recognized by the World Health Organization (WHO) and other international organizations for its reliable clinical efficacy for many years [1]. With the rapid development of modern medical technology, researchers have gradually shifted from focusing on the linear relationship between “effect and intervention” to examining the neurological relevance or other biological mechanisms of acupuncture. In the TCM culture, the meridian system is a systematic order of empirical knowledge that functions as the basis of acupuncture treatment. A large number of known or unknown acupoint nodes are scattered on the body surface with the meridian system as the carrier. However, acupoint specificity and meridian system have always evoked controversy among international scholars in related fields. As for acupoint specificity, some scholars consider that there are differences in the efficacy between acupoint-based acupuncture and non-acupoint-based acupuncture. Therefore, in recent years, a large number of researchers have carried out in-depth studies on acupoint specificity from multiple perspectives, including literature, clinic, biological structure, and biophysical properties [2, 3]. For example, they tried to analyze the influence of acupoints (PC6 [4], LI4 [5], KI1 [6]) on the human body by studying the activity characteristics of EEG signals, and the results showed that all these acupoints could activate specific cerebral cortex regions. Moreover, other studies on acupoint specificity include acupoint temperature characteristics [7] and lateralized specificity [8]. Unlike the nervous system, endocrine system, or immune system, the debate over the meridian system has focused on the lack of anatomical evidence. However, some studies have shown that these meridians, which carry important acupuncture points, are densely populated with nerve endings, nerve receptors, capillaries, mucopolysaccharides, and mast cells [9, 10]. It is worth mentioning that Benias et al. in their latest study found a previously unrecognized but widespread and macroscopic space within and between tissues, which expanded and standardized the concept of the human interstitium [11]. The interstitium is a complex network of fluid-filled channels, and its location, form of existence, and potential function (which may help explain the mechanism of cancer spread) have made many scholars associate it with the meridian system in the field of TCM. Although the correlation between them needs to be further analyzed and proved, by comparing the structural characteristics of interstitium and meridian system, it is undeniable that the network paradigm has always been the basis for the body to transmit information and control the multisystem cooperation.

Modern medicine regards the human body as a large-scale network composed of multifunction systems. The systems are closely connected through their common language. From the macro perspective, any abnormal event of physiological function is fed back by multiple network nodes. The authors in [1] consider that acupuncture treatment is a comprehensive regulatory process of the network. Regulatory information is cascaded and amplified by the acupoint network activated by physical stimulation, finally acting on diseases depending on the distribution characteristics of the meridians system. With the rapid development of network science, it has become a new trend in TCM research to explore the relationship between acupoints and diseases with network thinking. Network thinking is concerned with the interaction between the internal components of the system, to clarify the structure, function, and evolution of the system. Different from traditional multisource statistical analysis, the network analysis methods can provide new theoretical support for acupoint-disease research, including a visual schematic diagram, quantitative index of the overall or local topology of the network, and a mathematical model for acupoint-disease network (ADN) [12]. For instance, the authors in [13] established a complex network routing model of meridians, proving that the basic function of meridians is to transmit and regulate physiological information. The authors in [14] indicate that acupoint selection for different diseases is complex in terms of structure by establishing a mapping network of 233 diseases and 232 acupoints. Furthermore, a large number of scholars analyzed the acupoint selection rules of specific diseases with the complex network paradigm, including simple obesity [15], brain system [16], and heart system [17]. It is worth mentioning that the authors in [18] established a multidimensional network model (unweighted and undirected) based on the relationship between acupoints and diseases and proved that ADN has complex network properties by analyzing the static characteristics of the network (high clustering, scale-free, and small-world characteristics), which provided us with the theoretical basis to explore key acupoints of human body by using complex network.

There are always some key nodes with great influence in various complex networks. In general, the influence of nodes can be highlighted from the topological structure of the network model or the location attribute of nodes [19, 20]. For ADN, stimulation of a single acupoint node cannot improve all symptoms because symptoms involve multiple system dysfunctions and multiple targets. Meanwhile, the area that each acupoint can activate and regulate is also different. Therefore, researchers pay more attention to the synergistic effect of acupoint combination [21] and try to find the optimal acupuncture prescription for specific diseases based on this effect [22, 23]. For example, the authors in [24] used artificial neural network (ANN) to train the relationship between symptom information and selected acupoints, selected 11 key nodes with the highest average precision score, and systematically described acupoint indications through data mining technology. It can be seen that the key nodes in ADN are not isolated functional nodes, but some core nodes with high influence, wide range of regulation, and multiple combination modes.

In this paper, we firstly constructed a weighted undirected acupoint-disease network based on clinical acupuncture prescription literature and obtained reliable nodal centrality parameters by counting the acupoint selection frequency of prescription. Note that the acupoint-disease network is a one-dimensional homogeneous network, which is based on the synergistic effect of acupoints on disease. Secondly, we proposed a key node mining method based on the meridian system. Considering the intersection of acupoint functions, the proposed method balances the contribution of nodes in each community to the ADN on the basis of the meridian system. After preliminary screening, we designed a multiconstraint key node evaluation index based on the static characteristics of the network and marked a set of key acupoint nodes with high influence according to the node scores. We consider that key acupoints play an important role in the coordination of multiple physiological systems. They will be used as the core acupoints in the acupuncture treatment process to help TCM doctors find the optimal acupoint combination for specific diseases under resource constraints.

## 2. Materials and Methods

### 2.1. Data Availability

The data source of this paper consists of two parts. The first part is the statistical results of the acupoints frequently cited in the acupuncture prescriptions for common diseases, which is the summary of the acupuncture prescriptions for common diseases recorded in 5733 Chinese Clinical Acupuncture literature [25]. The data source involved a total of 50 common human diseases, including neurological, respiratory, digestive, cardiovascular, dermatological, gynecological, and a variety of rehabilitation diseases. These common diseases are widely representative of the therapeutic goals of TCM acupuncture. Specifically, taking diarrhea as an example, the authors claimed that there were 90 prescription acupoints related to the symptom, and the cumulative number of selected acupoints was 737, among which the 10 most frequently used acupoints were ST36, ST25, CV8, GV7, GV1, CV4, PC6, BL20, CV6, and BL25. We used the 10 acupoints with the highest frequency as acupoint nodes with the strongest ability to regulate diarrhea symptoms, to establish a fully connected subnetwork based on a single disease. The second database is used for calibration, which is the planning textbook for Chinese colleges of traditional Chinese medicine [26]. This dataset is used to demonstrate whether the selected acupoints are still relevant to the specified symptoms in the latest studies. If the information conflicts, the acupoint is discarded and the number of subnetwork nodes is reduced by 1. In summary, the ADN is established based on 50 common diseases and 135 acupoints involved in the data source. The original data can be downloaded according to the citation, and the preprocessed node and edge files are available from the corresponding author on reasonable request.

### 2.2. Construction of ADN

In this paper, the ADN is an acupoint association network based on the synergistic effect of acupoints on disease. It is abstracted as a graph *G*=(*V*, *E*), where *V* is the set of acupoint nodes and *E* is the set of edges. It is worth mentioning that there is an edge between two acupoint nodes if they can have a synergistic effect on the same disease. When the two acupoints have a synergistic effect on multiple types of disease, the connection between the two is even stronger, which is reflected in the weight of the edge. By counting the number of diseases directly affected by each pair of acupoints, we can obtain the statistically based edge weights (0 ≤ *w*_*ij*_ ≤ 50). The number of nodes in the graph *G* is denoted as *N*=|*V*|, and the number of edges is denoted as *M*=|*E*|. In mathematics, the network is usually represented by an adjacency matrix. For a network *G*=(*V*, *E*) with *N* nodes, its adjacency matrix *A*={*a*_*ij*_}_*N*×*N*_ is an *n*-order square matrix, where *a*_*ij*_ represents the element of the *i*th row and the *j*th column of *A*. Since ADN is a weighted undirected network, *a*_*ij*_ is as follows:

(1) $$\begin{array}{c} a_{ij}=\left\{\begin{array}{cc} w_{ij}, & \text{there is an edge with weight }w_{ij}\text{ between }v_{i}\text{ and }v_{j},\\ 0, & \text{there is no edge between }v_{i}\text{ and }v_{j}. \end{array}\right. \end{array}$$

**Figure 1:**
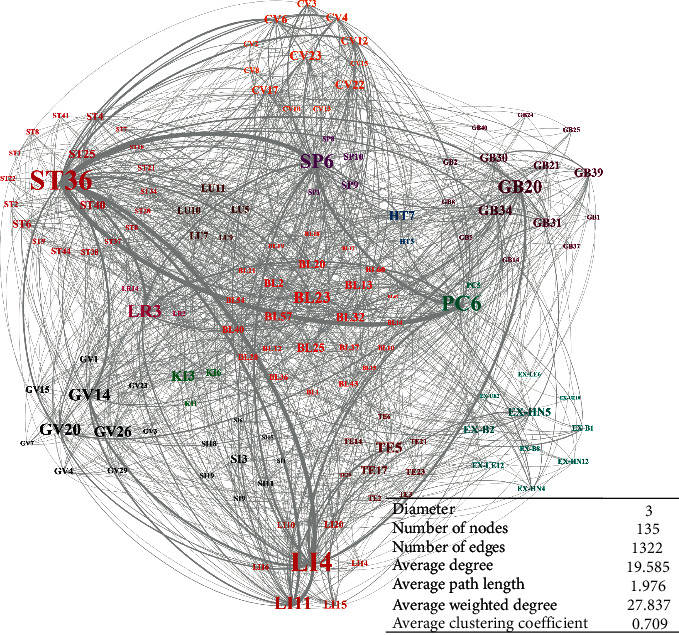
Network model of ADN and its static characteristic parameters. In the network model, the width of the edge reflects different weights, and the color of the node label indicates that the acupoints are in different communities. The way of community division is explained in Section 2.3.1.

Figure 1 shows the network model of ADN and its static characteristic parameters. It can be seen that the ADN conforms to the network mechanism of various complex network models in statistical properties [27].

### 2.3. Key Acupoint Node Mining Based on Meridian Distribution Characteristics

The key nodes refer to a few special nodes that play an important role in the structure and function of the network compared with ordinary nodes in the network. In the case that the transmission, synchronization, and control mechanism of acupoints to physiological information cannot be determined from the perspective of modern medicine, the highly abstract complex network model provides us with an effective means to explore key acupoints from the micro perspective (nodes and links). In this work, we combine the topological structure of ADN with the actual meridian distribution to construct a node mining method that meets the meridian specificity. In this paper, the definition of the key acupoint is that the acupoint or the prescription with the acupoint as the core has a wide spatial distribution of indications. The steps of key acupoint node mining include (1) dividing the community based on the distribution characteristics of meridians; (2) selecting candidate nodes in each community; and (3) selecting key acupoint nodes in the candidate set according to the proposed key node evaluation index.

#### 2.3.1. Community Division Based on the Meridian System

The community division of ADN is to divide the 135 nodes involved in the data source strictly according to the meridian system. Actually, the community division is a process of classification according to acupoint attributes. There are 14 meridians in the human body, of which 12 meridians are symmetrically distributed in the head, trunk, and limbs. The remaining two meridians (called conception vessel and governor vessel) are located in the anterior and posterior midline of the body. In addition to being evenly distributed on the 14 meridians, some auxiliary acupoints are still scattered around the surface of the body, which are uniformly known as the extraordinary points. We divided the nodes according to the location attributes of acupoints, i.e., 15 communities.

There are two main reasons for classification before node evaluation. First, it is necessary to avoid focusing all the key nodes on some special meridians. Since the position of each meridian is different, acupoints on some meridians may have the ability to regulate multiple symptoms in a local area. The authors in [28] proved that the spatial distribution of acupoint indications is wide, and the specificity of acupoint indications has always been an important research direction in the field. Meanwhile, the acupuncture has the principle of “point selection along the affected meridian,” which indicates that different acupoints on the same meridian have a wide range of function intersections. Therefore, the community division mode based on the meridian system can highlight some important acupoints with specific curative effects in the network model and balance the contribution of nodes in each community to the overall network, to ensure that TCM doctors always have core acupoints for reference in each target meridian after diagnosis. Second, in order to improve the reliability of the key node set, we need to pay attention to the extraordinary points involved in the data source. The extraordinary points refer to a series of acupoints with names, fixed positions, and main indications. In our data source, some extraordinary points can work synergically with acupoints of fourteen meridians on a variety of common diseases, which means that the importance of the extraordinary points in the network cannot be ignored. In sum, the ADN is divided into 15 communities according to the location attributes of acupoints, including fourteen meridians and extraordinary points. Each community is shown in different colors in Figure 1.

#### 2.3.2. Selection of Candidate Nodes

To improve the reliability of the key node set, we preliminarily screened the nodes in each community. Making a candidate node set is a common step in node mining. In general, the candidate set is usually composed of the top 10% nodes of each community according to a certain evaluation index and the nodes connecting different communities [29]. However, in our work, the ADN is composed of 50 fully connected subnetworks (according to the edge-building principle, a small-scale network of full connection will be formed between prescription acupoints based on a single disease type, which is called a full connection subnetwork), and nodes within each community may belong to multiple subnetworks. Therefore, we ignored the nodes connecting different communities in the process of making the candidate set, to maintain the limited and reliable dimension of the candidate set. According to the network size and the community number of ADN, each community will select four nodes to form a candidate set with no more than 60 dimensions. It is worth mentioning that not every community has at least four prescription acupoints in the data source. At this point, all nodes in the community are added to the candidate set. For example, the data source only involves two prescription acupoints (PC6 and PC5) in the pericardium meridian of hand-Jueyin (PC), so both are candidate nodes.

In general, the evaluation index of candidate nodes is usually a centrality parameter of the node. Classical centrality methods include degree centrality and betweenness centrality. On this basis, a large number of scholars proposed improved centrality methods, such as WFCA [30], GFT centrality [31], ECP centrality [32], and eccentricity centrality [33]. These methods can accurately quantify the importance of nodes in the network from multiple perspectives, such as target node attribute, neighbor node attribute, and path attribute. However, the ADN is a small-scale scale-free network with strict meridian topology constraints, which means that the low-resolution or local-topology-based centrality method is not suitable for our work. Therefore, we choose the classical closeness centrality as the evaluation index of candidate nodes. Closeness centrality is one of the basic concepts in topological space, which is strongly dependent on the global topology of the network. For an undirected connected graph, the closeness centrality of a node is most naturally defined as follows:

(2) $$\begin{array}{c} C_{C}\left(v_{i}\right)=\frac{N-1}{\sum_{j=1,j\neq i}^{N}d_{ij}}, \end{array}$$

where *d*_*ij*_ is the length of the shortest path from node *v*_*i*_ to node *v*_*j*_, and *N* is the network size. The larger the *C*_*C*_(*v*_*i*_) is, the closer the node is to the center of the network, that is, the more important it is in the network.

#### 2.3.3. Selection of Key Acupoint Nodes

**Figure 2:**
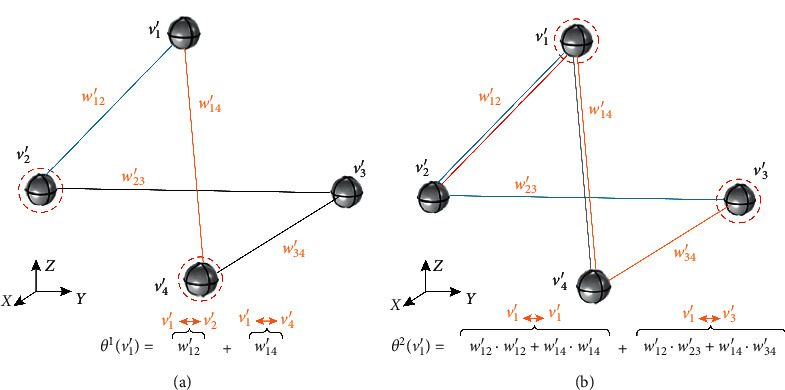
The weighted influence of node *v*_1_′ in *G*′: (a) 1-hop path; (b) 2-hop path.

Quantifying the importance of a node to a network often involves complex calculations with multiple constraints. Choosing practical constraint conditions is helpful to improve the reliability and accuracy of the quantization method. We consider that the importance of a node can be judged by its influence on all nodes in the network. For a weighted undirected network, paths of different lengths have different contributions to nodes. Meanwhile, the weight on each path determines the influence of the path. Based on the above two principles, we designed a key node evaluation index with high specificity for ADN. Considering the principle of acupoint selection and the dimension of the candidate set, the key acupoint node set will be composed of the two nodes with the highest score in the candidate set of each community. In other words, the key acupoint node set includes a total of 30 nodes from 15 communities. The evaluation index of key acupoint nodes is as follows:

(3) $$\begin{array}{c} \text{Key}\_\text{node}\left(v_{i}\right)=\sum_{k=1}^{D}\frac{\theta_{i}^{k}}{\theta^{k}}, \end{array}$$

where *θ*_*i*_^*k*^ represents the weighted influence of node *v*_*i*_ on all nodes in the network in the path of length *k*; *θ*^*k*^ represents the accumulation of the weighted influence of all paths of length *k* in the network; and *D* is the network diameter, which refers to the maximum value of the shortest distance between any two points in the network. It can be seen that Key_node(*v*_*i*_) represents the proportion of the weighted influence of node *v*_*i*_ on the global network to the total value of the network within the range of *D*-hop path. In ADN, this means that the index pays more attention to the disease regulatory ability (including regulatory range) of acupoint combinations involving node*v*_*i*_. To facilitate understanding, we take the simple network *G*′ with *N*=4 as an example to illustrate the calculation process of *θ*_*i*_^*k*^ (the connection of nodes is random). The network model of *G*′ is shown in Figure 2. According to the network model, the diameter *D*′ of *G*′ is 2. When discussing the importance of the node *v*_1_′ in the network, we need to calculate the weighted influence of node *v*_1_′ on the network in the 1-hop path and the 2-hop path, respectively. It can be seen from Figure 2(a) that the weighted influence based on the 1-hop path is actually the weighted degree of node *v*_1_′, which reflects the closeness of the connection between the node *v*_1_′ and its neighbor nodes. In our network construction rules, neighbor nodes refer to multiple prescription acupoints with a direct therapeutic effect on a certain disease, so the weighted influence of 1-hop path reflects the ability of node to directly participate in physiological regulation. Similarly, Figure 2(b) shows the weighted influence of node *v*_1_′ on the network in the case of 2-hop path. In acupuncture theory, “distant acupoint selection” will actually have the possibility of regulating more types of symptoms, which is reflected in the number of connection modes established between two nonneighbor nodes due to different symptoms. Different from the 1-hop paths, the selection of multihop paths will include repeated paths between neighbor nodes. We consider that this process not only helps to strengthen the influence of the combination of neighbor nodes in the whole network, but also reflects the regulation ability of different paths caused by the change of intermediary node among nonneighbor nodes. In other words, even though the computational logic is based on the global topology, the nodes with higher degree centrality still have a greater contribution to the ADN (reflecting the influence of the local topology on the global topology).

Since ADN is an undirected network, the adjacency matrix of the graph *G*=(*V*, *E*) is a real symmetric matrix. Meanwhile, one of the properties of the adjacency matrix (weighted) is that the element in its *k*-power represents the weight accumulation of all paths of length *k* between two nodes. That is,

(4) $$\begin{array}{c} \theta_{i}^{k}=\sum_{m=1,m\neq i}^{N}A^{k}\left(v_{i},v_{m}\right),\\ \theta^{k}=\frac{1}{2}\sum_{n=1,m=1,n\neq m}^{N}A^{k}\left(v_{n},v_{m}\right). \end{array}$$

Therefore, the evaluation index of key acupoint nodes is as follows:

(5) $$\begin{array}{c} {\text{Key}}_{\text{node}\left(v_{i}\right)}=2\sum_{k=1}^{D}\frac{\sum_{m=1,m\neq i}^{N}A^{k}\left(v_{i},v_{m}\right)}{\sum_{n=1,m=1,n\neq m}^{N}A^{k}\left(v_{n},v_{m}\right)}. \end{array}$$

## 3. Results

After community division, a total of 55 nodes make up the candidate set according to the sorting results of centrality parameters. By comparing Key_node of the candidate nodes, we can obtain the two key acupoint nodes with the highest score in each community. The screening process not only keeps the classic pattern of node mining (to ensure the accuracy of the results), but also avoids the traversal of the global network, which can greatly reduce the computational complexity with the increase of data sources.  Table 1 shows the community attributes and centrality parameters of key acupoints. It is worth mentioning that candidate nodes with higher *C*_*C*_(*v*_*i*_) do not necessarily have higher Key_node(*v*_*i*_). For example, CV4 is the third candidate in the CV community (0.568 for *C*_*C*_(CV23) and 0.552 for *C*_*C*_(CV22)), but CV4 had the highest Key_node score in the community. It is shown that the proposed index can not only combine the global topological characteristics of the network in the same way as the closeness centrality, but also overcome the problem that the latter cannot analyze the node relations by combining the weights of edges.

**Figure 3:**
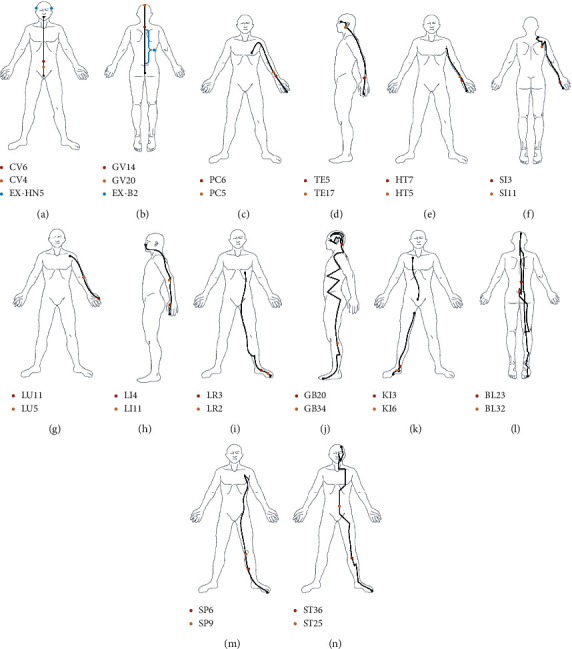
Spatial distribution of key acupoints: (a) conception vessel, (b) governor vessel, (c) pericardium meridian of hand-Jueyin, (d) triple energizer meridian of hand-Shaoyang, (e) heart meridian of hand-Shaoyin, (f) small intestine meridian of hand-Taiyang, (g) lung meridian of hand-Taiyin, (h) large intestine meridian of hand-Yangming, (i) liver meridian of foot-Jueyin, (j) gallbladder meridian of foot-Shaoyang, (k) kidney meridian of foot-Shaoyin, (l) bladder meridian of foot-Taiyang, (m) spleen meridian of foot-Taiyin, and (n) stomach meridian of foot-Yangming.

Figure 3 visualizes the spatial distribution of key acupoints. It can be seen that the key acupoints are uniformly distributed in the meridian system. This is an interesting result because it may help to explore the relationship between acupoint indications and acupoint specificity. After the diagnosis pattern and the prescription meridian are determined, the key acupoints on the meridian can improve the stability and accuracy of the prescription effect. At this point, there are always locally optimal acupoints to choose when exploring the ideal acupoints in the local indicative area. Besides, we calculated the proportion of these key acupoints in common disease prescription acupoints and total acupoints in human body, which were about 22.2% and 7.3%, respectively. The core degree of each acupoint in the prescription acupoint can be reflected by the Key_node value, as shown in Table 1.

**Figure 4:**
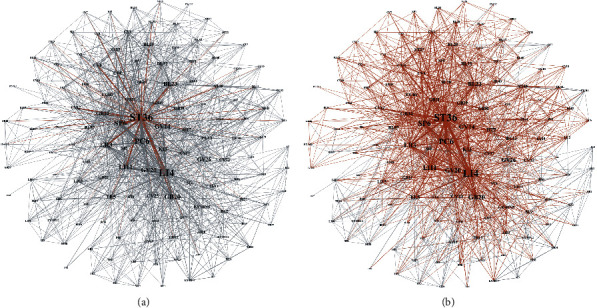
The regulation range of 1-hop acupoint combination of key acupoints in ADN: (a) ST36; (b) all 30 key acupoints.

In addition, in order to verify the centrality of key nodes in the network, we used the static parameters of the network to quantitatively analyze the influence of key nodes on ADN from the perspective of graph theory. It can be seen from Table 1 that ST36 has the highest value of Key_node. When ST36 is activated, a large number of acupoint nodes in the network can directly regulate multiple types of diseases through interaction with ST36 (within the 1-hop range).  Figure 4(a) shows the regulation range of the 1-hop acupoint combination of the activated ST36 to the whole ADN, and the red part shows the affected area. The statistical results show that the number of nodes that can cooperate with ST36 accounts for 68.9% of the network scale. When all 30 key nodes are activated, the number of acupoint nodes that can be directly combined within the 1-hop range accounts for 96.3% of the network scale, as shown in Figure 4(b). It can be seen that the key acupoint node can be used as the root node to improve the efficiency and accuracy of research when exploring the optimal acupoint combination for the treatment of some local diseases.

## 4. Performance Evaluation and Discussion

In order to verify the reliability of the key node set, we will adopt the three indexes of resolution, network loss, and accuracy, respectively, to evaluate the experimental results. Meanwhile, six classical node importance evaluation indexes are used in comparison experiments to show the advantages of the proposed algorithm in the aspects of network connectivity, topology structure, and weight benefit. The methods are degree, closeness centrality (CC), betweenness centrality (BC), eigenvector centrality (EC), k-shell algorithm, and clustered local degree centrality (CLD). It is worth mentioning that k-shell algorithm is a classic node influence definition method based on global network, which can effectively highlight nodes with small degree but great actual influence [34]; CLD algorithm comprehensively considers the local topological properties of nodes and their nearest neighbors and highlights the propagation ability between neighbor nodes by combining the clustering coefficients, which is a new important node mining method [35].

### 4.1. Resolution

Resolution is a common index to measure the performance of algorithms, which can accurately distinguish the mining precision of each algorithm for nodes with highly similar centrality in large-scale networks [36]. In order to accurately compare the resolution index of each algorithm, we scored all 135 nodes involved in the data source and compared the granularity of all scoring results. The resolution *f*(*r*_*A*_) is calculated as follows:

(6) $$\begin{array}{c} f\left(r_{A}\right)=1-\frac{\sum_{i=1}^{R}N_{i}^{2}}{R*N^{2}}, \end{array}$$

where *r*_*A*_ represents the sorting results of each algorithm, *N* represents the network size, *R* represents the granularity of the sorting results, and *N*_*i*_ represents the number of nodes owned by the *i*th type of results. It can be seen that when *R*=1, *f*(*r*_*A*_)=0; that is, the sorting result cannot distinguish the importance degree of nodes in the network. When the sorting result has fine granularity, the resolution of the algorithm will gradually approach 1 with the increase of the network size.

To facilitate the comparison of algorithm performance, we define Δ(*r*_*A*_)=1 − *f*(*r*_*A*_) to represent the resolution index. The smaller the Δ(*r*_*A*_) is, the better the resolution of the algorithm is.  Table 2 shows the comparison results between the proposed algorithm and the six known algorithms. It can be seen that the proposed algorithm has a resolution similar to EC and CLD and has more prominent advantages in precision than the classical k-shell algorithm and other centrality methods.

**Figure 5:**
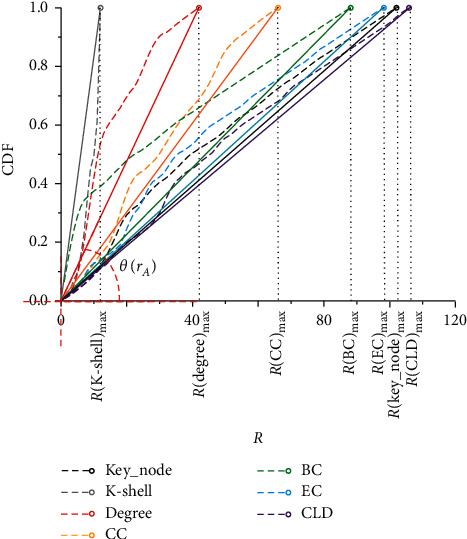
The CDF curve of the resolution experiment.

In addition, in order to make a concise comparison between the proposed algorithm and the existing algorithms, we can draw the Cumulative Distribution Function (CDF) curve as shown in Figure 5. The *x*-axis in the figure represents the granularity of each sorting result, and the *y*-axis represents the cumulative percentage of the number of nodes in the sort result. The resolution of an algorithm can be quickly concluded by observing the slope of the line between *R*(*r*_*A*_)_max_ and the origin of the coordinate system (or the angle *θ*(*r*_*A*_) between the line and the *x*-axis). The smaller the *θ*(*r*_*A*_) is, the higher the resolution is. Experimental results show that the proposed algorithm has a higher resolution performance, similar to EC but slightly lower than CLD. For EC and CLD algorithms, since EC evenly distributes the score of the target node to its neighbors and assigns a new score to the target node in each iteration, almost every node in the network has a score that is different from the other nodes when the calculation is finished. Meanwhile, the CLD method can distinguish the importance of nonsparse network nodes well. Similar to EC, CLD combines the clustering coefficient of the target node with the topological characteristics of the nearest neighbors, to obtain high-precision sorting results. In conclusion, the proposed algorithm has a similar resolution to the EC and CLD algorithm with excellent performance, which can meet the requirements of key node mining in ADN.

### 4.2. Network Loss

We consider that the key acupoint, as an important entry point to regulate the local physiological state, plays a role of a bridge in ADN. It can ensure the reasonable, robust, and accurate transmission of physiological information between target regions. In other words, each key acupoint node plays a decisive role in the connection of the whole network. Therefore, we will evaluate the key nodes by the node deletion method and illustrate the advantages of these nodes in network connectivity by comparing the network loss with the other six algorithms.

The node deletion method indicates that the deletion of a node will affect the connectivity of the network in two ways [37]. On the one hand, the deleted node cannot keep the connectivity state between the node and other nodes, which is called direct loss (DLOS). On the other hand, the remaining nodes may no longer connect due to the loss of bridge nodes, which is called indirect loss (ILOS). The unit loss caused by node deletion is inversely proportional to the reciprocal of the shortest distance from the node to other nodes. By taking the unit loss as the weight and summing up the disconnected nodes, we can clearly quantify the impact of node deletion on network connectivity. According to the above definition, DLOS and ILOS are as follows:

(7) $$\begin{array}{c} \text{DLOS}\left(v_{i}\right)=\sum_{j=1,j\neq i}^{N}\frac{1}{d_{ij}},\\ \text{ILOS}\left(v_{i}\right)=\sum_{l=1,l\neq i}^{N-1}\sum_{j=l+1,j\neq i}^{N}\frac{\delta \left(v_{l},v_{j}\right)}{d_{lj}}, \end{array}$$

where *d*_*ij*_ represents the shortest distance from node *v*_*i*_ to node *v*_*j*_; *N* represents the size of the network; and *δ*(*v*_*l*_, *v*_*j*_) has different values after deleting node *v*_*i*_, which is specifically defined as follows:

(8) $$\begin{array}{c} \delta \left(v_{l},v_{j}\right)=\left\{\begin{array}{cc} 0, & v_{l}\text{ and }v_{j}\text{ are still connected,}\\ 1, & v_{l}\text{ and }v_{j}\text{ are not connected.} \end{array}\right. \end{array}$$

Therefore, the total loss caused by deleting node *v*_*i*_ to the network is

(9) $$\begin{array}{c} \text{TLOS}\left(v_{i}\right)=\text{DLOS}\left(v_{i}\right)+\text{ILOS}\left(v_{i}\right)=\sum_{l=1}^{N-1}\sum_{j=l+1}^{N}\frac{\delta \left(v_{l},v_{j}\right)}{d_{lj}}. \end{array}$$

In addition, the network loss can be maximized for the central node of the star network with the same network size. The influence of network size can be eliminated by normalizing TLOS(*v*_*i*_) with the maximum value, and the result is as follows:

(10) $$\begin{array}{c} C\left(v_{i}\right)=\frac{4\text{TLOS}\left(v_{i}\right)}{\left(N-1\right)\left(N+2\right)}. \end{array}$$

In ADN, since the proposed method adds location attributes based on meridian system to nodes, all comparison algorithms select key node sets with the same dimension in each community according to the same community division method before performance evaluation. Finally, the key acupoint set *U* output by each algorithm and the corresponding network loss *C*(*U*) are shown in Table 3.

Limited by the number of communities and network size, the key node sets of the seven algorithms are very similar. However, the output results of the proposed method still have higher network loss, which is close to CC but lower than degree. This is because the node deletion method has a strong correlation with degree centrality, which is manifested in the fact that the neighbor node of the deleted node is given a large weight. (In the proposed algorithm, we also emphasize the influence of local topology on the network, which is explained in 2.3.3. Therefore, we are not surprised by the results of the comparison with degree centrality.) In addition, since the proposed method selected candidate nodes according to CC, it is possible to obtain network loss parameters similar to CC when the network size is limited. In view of scientific rigor, we will continue to expand experimental data sources in future work, to avoid similar evaluation results caused by minor differences in experimental results. Nevertheless, in terms of network connectivity, the output results of the proposed method have more significant influence than those with higher resolution performance, such as CLD and EC.

### 4.3. Accuracy

In this section, we will evaluate the accuracy of the key node set by measuring and comparing the correlation between a set of benchmark score results and the experimental results of each algorithm. A higher correlation means that the sorting results of node importance are more accurate. However, the ADN is a weighted network, so the benchmark scoring criteria need to introduce a variety of attributes of network nodes, including the connection relation between nodes, the topological characteristics of the network, and the strength of the interaction between nodes. Therefore, we adopted the weight reduction method as the benchmark scoring criteria for ADN nodes [38]. The weight reduction method is a basic method to evaluate the influence of nodes on a complex network, which has a comprehensive evaluation perspective but rough evaluation ability. It focuses on two aspects of the network, including the weighted degree of nodes and the location attributes of nodes in the network. The higher the weighted degree of the node, the closer the connection with other nodes. The more important the location of the node, the shorter the path through the node, which also verifies the contribution of the node to the network connectivity from another perspective.

**Figure 6:**
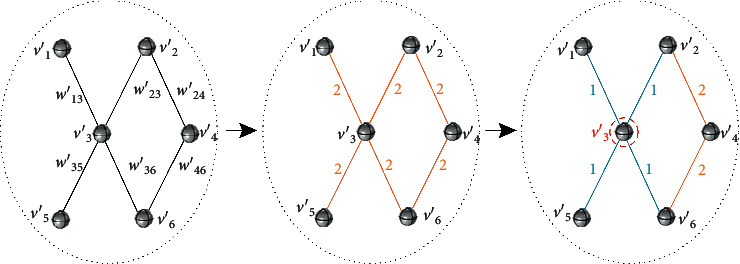
The analysis process of node location attribute in the weight reduction method.

When analyzing the location importance of node *v*_*i*_, the weight reduction method sets the weight of every edge in the network as 2 and reduces the weight of all the edges within the 1-hop range of the node to 1.  Figure 6 shows the analysis process of node location attribute (schematic). At this point, the weighted average distance of the network will change. If the location of the node is important, the weighted average distance of the network will become smaller. Let *s*_*i*_ represent the weighted degree of the node and *l*_*i*_ represent the weighted average distance of the network after the weight of the adjacent edge of *v*_*i*_ is set to 1; then the benchmark scoring criteria are as follows:

(11) $$\begin{array}{c} W\left(v_{i}\right)=\frac{s_{i}}{l_{i}}. \end{array}$$

**Figure 7:**
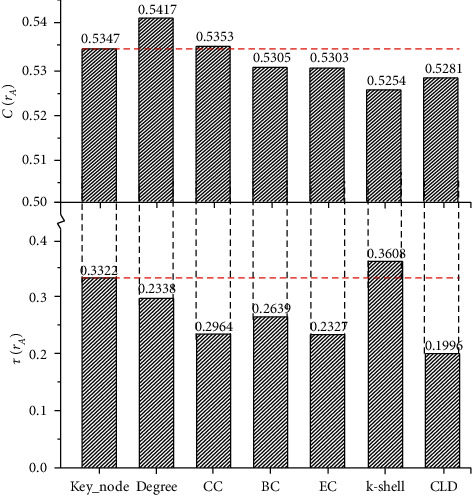
Performance comparison of sorting results of the seven algorithms.

The classical Kendall *τ* correlation coefficient in statistics is used for correlation measurement, which is used to represent the ordinal correlation between two measurement quantities [39].  Figure 7 shows the accuracy of the node importance ranking results of the seven algorithms. It can be seen that the sorting result of the proposed algorithm has a higher correlation coefficient with the benchmark, which is far greater than that of EC and CLD with higher resolution, and degree and CC with higher network loss. Although these comparison algorithms obtain sufficient accurate node scores from different perspectives of network topology, they still lack consideration of weight benefit when comprehensively analyzing the node importance of the weighted network. In addition, k-shell method obtained the highest correlation in this experiment, but from the calculation process of the algorithm, k-shell algorithm only classifies the location attributes of nodes in coarse granularity. A large number of nodes with the same score have no additional parameters to subdivide their importance degree. The sorting of nodes in different levels will affect the analysis results of correlation. Therefore, we still need to combine the resolution to evaluate the accuracy of each result.

### 4.4. Discussion

As a way of thinking that can explain the underlying network phenomenon and its complexity, network science provides us with a new dimension to understand the role of acupoints in the correlation, control, and coordination of physiological information. In order to explore the important acupoints in complex meridians, we designed a highly specific key node mining method based on ADN. In the three groups of performance evaluation experiments, the proposed method shows good performance, which makes it more suitable for the important node mining of ADN than other known centrality methods. Except for some special extraordinary points, key acupoints are evenly distributed in the fourteen meridians of the human body (the data source does not involve Ashi acupoints). We have made statistics on the types of diseases that can be directly affected by all the key acupoints in the data source, of which the most is ST36 (36 diseases), and the average number is 8.97. It can be seen that external stimulation on key acupoints can affect more physiological states compared with common acupoints. By taking advantage of this characteristic, TCM doctors can select the points with high influence on the target meridians after diagnosis. Therefore, although the acupuncture prescription chosen by the TCM doctor may vary depending on different prior experience, these key acupoints always play the role of core acupoints. They can be the first choice of various acupoint selection methods, such as “point selection along affected meridian” or “point selection on related meridians,” and provide TCM doctors with a wider range of physiological regulation and more possible acupoint matching modes. Our work is to mark these key acupoints accurately so that TCM scholars can optimize and perfect acupuncture prescriptions for common symptoms.

#### 4.4.1. About the ADN

The ADN is a one-dimensional network. The connection between acupoint nodes was established through the synergistic effect on different diseases. On the one hand, with the continuous development of TCM theory, although some Ashi acupoints have been marked and classified into new extraordinary points, the types and distribution of acupoints (especially the acupoints on the fourteen meridians) are almost unchanged. On the other hand, the synergistic effect of acupoints on diseases is the result of the long-term practice and summary of TCM acupuncture culture. Therefore, for the ADN, both nodes and edges are extremely stable, which is different from many dynamic network models. Moreover, the TCM theory [26] indicates that the meridian system is a systematic order of empirical knowledge that functions as the basis of acupuncture treatment. Therefore, the principle of acupoint selection is closely related to the distribution of meridians. In ADN, in order to reflect the connection between acupoints, meridians, and diseases, we divided the nodes of the network into communities based on the meridians system, as shown in Figure 1. Finally, 30 key nodes were evenly distributed in 15 “communities” of the human body to highlight the influence of meridians on acupuncture treatment and acupoint specificity.

#### 4.4.2. About the Key Acupoint Set

As for the key acupoints, we can discuss them from the perspectives of network paradigm and TCM clinical acupuncture.

From the perspective of the complex network, key acupoint nodes from different communities coupled discrete to unrelated acupuncture prescriptions. Key_node quantifies the influence of acupoint nodes on the network through weighted accumulation in multihop paths, which also provides the possibility for each node to obtain an independent and discriminative individual evaluation. During the evaluation process, the influence of nodes on network connectivity is highlighted by traversing different paths in the network. Although the importance of nodes within the two parallel communities is not differentiated, the high-resolution scoring results can still help us analyze the contribution of each node to network connectivity at a fine-grained level. In addition, we pay more attention to the specificity of acupoints to common diseases. 50 fully connected subnetworks can be constructed with the disease types involved in the data source as the edge-building principle. The overlapping edges represent the correlation strength between a pair of nodes in the form of weights. Meanwhile, the node pair can improve their value in the network by sharing the weight benefit of the edge. A high value means that the key acupoint nodes have a larger range of disease regulation and more acupoint combination modes. This node scoring method meets our requirements for target acupoints; that is, the key acupoint nodes can be used as core acupoints to regulate the local physiological states to help researchers explore targeted and high-impact acupoint combinations.

From the perspective of TCM clinical acupuncture, we can understand the key acupoint set from three aspects. Firstly, in general, a certain acupoint can be selected specifically for a particular disease, and it has a specific indication for the disease. However, the specificity of acupoint indication and the specificity of acupoint selection are not always identical. The authors in [40] demonstrate that the selection of an acupoint for a particular disease does not imply that the acupoint has specific indications for that disease. In other words, in the two-dimensional acupoint-disease network, the nodes are not bidirectional. Therefore, we visualized acupoint relationships based on the network paradigm through different symptoms. This one-dimensional network helps to understand the macroscopic connections between acupoints from the point of view of symptoms, so as to carry out the body cognition based on statistics. At this point, a wide range of prescription types helps to illuminate the overall robustness of the human system. In this sense, we are committed to exploring key acupoint nodes based on the whole system. These well-regulated acupoints will be given a higher weight when researchers analyze local symptoms, to determine the best acupoint prescription faster and more accurately. Secondly, acupoint indications have specific spatial distribution rules [28]. For a long time, a large number of scholars have explored the relationship between common acupoints and symptom areas based on clinical data. However, traditional methods are more demanding for clinical data, such as obtaining mapping labels between the patient's prescription acupoints and the location of local symptoms. These label data with high labor costs are highly susceptible to subjective factors of patients. Therefore, exploring key acupoints from ADN can be understood as analyzing the abstract distance between acupoints from a large amount of clinical prescription data. In the Key_node scoring criterion, acupoints are associated with each other in the form of multiple hops. Obviously, the adjacent acupoints (one-hop pathways) have better synergy in regulating the physiological state. Meanwhile, the key acupoints were uniformly distributed in the 14 meridians by means of community division (except for two extraordinary acupoints), which means that the selected key acupoints were still guided by the meridians system, and the abstract distance between acupoints was considered based on the clinical prescription data. The disadvantage is that the analysis of key acupoints based on big data is for the whole body system, and the specific symptoms need to be modeled and discussed in a fine-grained way. We will discuss this in detail in the limitations of ADN. Finally, the key acupoints show stability in the deviation of the diagnosis pattern. The diversity is the result of the particular decision-making requirements for acupuncture treatment, which include assembling clinical data and identifying diagnosis patterns to ensure the use of appropriate acupoints [41]. The choice of diagnosis pattern depends on the clinical experience and medical knowledge of individual doctors. Pattern deviation is inevitable, leading to different acupoint prescriptions used by different doctors. In the latest study, the author in [42] investigated acupoint prescriptions by diagnosis patterns using network and text mining analyses. The results identified common acupoints under different diagnosis patterns. We refer to Table 2 of the literature [42] to illustrate the clinical significance of the key acupoint set in different diagnosis patterns, as shown in Table 4. For the top five most frequent diagnoses, the authors calculated the five highest frequency prescribed acupoints, respectively. The results showed that ST36, LI4, and SP6 were frequently prescribed across all five diagnosis patterns. On the other hand, for each diagnosis pattern, the acupoints with the highest frequency are ST36, LI4, KI3, LR3, and ST36 in order. All of these acupoints are included in the key acupoint set of our study. It can be seen that the key acupoints show strong stability in the diagnosis pattern and the doctor's personal knowledge system. Therefore, in the process of selecting some prescription acupoints according to the symptom, it is of certain significance to give priority to the key acupoints to improve the accuracy of the prescription.

#### 4.4.3. Limitations of ADN

This study had certain limitations. First of all, ADN is an undirected network, which means ADN defaults to the corresponding relationship between acupoint selection and acupoint indication. As mentioned in literature [40], the selection of an acupoint for a particular disease does not imply that the acupoint has specific indications for that disease. Therefore, the specificity of acupoint indications inferred from clinical observation should be considered, which requires the combination of forward and reverse inference. In fact, ADN is the projection of two-dimensional acupoint-disease network on acupoint direction. For the reverse inference, we will model and quantify the disease nodes based on each acupoint attribute in the future work, so as to explore the corresponding relationship between acupoint selection and acupoint indication. Second, the spatial attribute of acupoints is fixed. Although the evaluation index of Key_node considers the abstract distance (synergy) between acupoints, the real spatial attribute of acupoints is not included in the evaluation constraint. In the complex network model, the spatial attribute of nodes can be captured by changing the edge weight calculation method. At this point, the *w*_*ij*_ is determined by the disease type and the relative distance of acupoints in the body. Finally, ADN will develop towards fine-grained research. One of the purposes of the interdisciplinary discussion on acupuncture prescriptions using network thinking is to understand the macroscopic physiological characteristics from the perspective of pervasive computing. Any disease is a challenge to the robustness of this complex system. Thus, statistically, a wide range of disease types in the data source contributes to the reliability of the critical acupoint set. However, in future studies, the ADN can continue to discuss specific disease or specific acupoint indication space, which will challenge the quantity and quality of data sources.

## 5. Conclusion

As an important component of traditional Chinese medicine, acupuncture has been proved effective for centuries. Meanwhile, as the basis of acupuncture, acupoint specificity has always been the focus of scholars in related fields. However, given the lack of anatomical evidence, international scholars have always been controversial about acupoint specificity. Therefore, it is necessary to find a new dimension to analyze the relevant characteristics of acupoints. Meanwhile, we found that some special acupoints frequently appeared in different clinical acupuncture prescriptions. Obviously, these acupoints play an important role in the coordination and control of the physiological system of the human body. They deserve to be marked in order to provide a reference for researchers in exploring the optimal acupoint combination for the treatment of common diseases. We consider that the optimization and perfection of acupuncture prescription will reduce the difference of acupoint selection caused by subjective factors, to improve the efficiency and effect of acupuncture therapy.

In the future work, we will continue to explore the human meridian and acupoint specificity based on the network paradigm. Although network thinking is different from traditional multisource statistical methods, its dependence on data is the same. The human body still has a large number of unknown acupoints, which also have independent position attribution and function. We will collect more reliable data sources, and on this basis we will (1) increase the scale of ADN to provide more references for TCM researchers, including new acupoints and their physiological regulation range, and (2) increase the constraint conditions of evaluation indexes for key acupoints (such as human symmetry) to further improve the reliability of key acupoints.

## Figures and Tables

**Table 1:** Community attributes and centrality parameters of 30 key acupoints.

Acupoint	Community	Closeness centrality	Key_node(*v*_*i*_) ∗ 100 (%)
TE5	Triple energizer meridian of hand-Shaoyang (TE)	0.586	9.36
TE17	Triple energizer meridian of hand-Shaoyang (TE)	0.550	4.10
HT7	Heart meridian of hand-Shaoyin (HT)	0.573	8.83
HT5	Heart meridian of hand-Shaoyin (HT)	0.480	1.53
SI3	Small intestine meridian of hand-Taiyang (SI)	0.532	4.21
SI11	Small intestine meridian of hand-Taiyang (SI)	0.502	2.25
LU11	Lung meridian of hand-Taiyin (LU)	0.541	3.99
LU5	Lung meridian of hand-Taiyin (LU)	0.528	3.54
LI4	Large intestine meridian of hand-Yangming (LI)	0.747	30.05
LI11	Large intestine meridian of hand-Yangming (LI)	0.630	19.87
PC6	Pericardium meridian of hand-Jueyin (PC)	0.665	24.54
PC5	Pericardium meridian of hand-Jueyin (PC)	0.524	3.30
GB20	Gallbladder meridian of foot-Shaoyang (GB)	0.619	15.02
GB34	Gallbladder meridian of foot-Shaoyang (GB)	0.581	10.26
KI3	Kidney meridian of foot-Shaoyin (KI)	0.589	7.40
KI6	Kidney meridian of foot-Shaoyin (KI)	0.516	3.09
BL23	Bladder meridian of foot-Taiyang (BL)	0.594	11.26
BL32	Bladder meridian of foot-Taiyang (BL)	0.557	7.03
SP6	Spleen meridian of foot-Taiyin (SP)	0.649	24.51
SP9	Spleen meridian of foot-Taiyin (SP)	0.541	6.20
ST36	Stomach meridian of foot-Yangming (ST)	0.764	38.94
ST25	Stomach meridian of foot-Yangming (ST)	0.554	7.26
LR3	Liver meridian of foot-Jueyin (LR)	0.622	18.35
LR2	Liver meridian of foot-Jueyin (LR)	0.512	2.35
CV4	Conception vessel (CV)	0.550	10.00
CV6	Conception vessel (CV)	0.547	6.45
GV14	Governor vessel (GV)	0.613	14.97
GV20	Governor vessel (GV)	0.605	12.35
EX-HN5	The extraordinary points	0.552	6.75
EX-B2	The extraordinary points	0.545	3.45

**Table 2:** Comparison results of the resolution index.

	Key_node	Degree	CC	BC	EC	k-shell	CLD
Δ(*r*_*A*_)	1.38*e*^−4^	1.13*e*^−3^	3.53*e*^−4^	2.92*e*^−4^	1.55*e*^−4^	1.67*e*^−2^	1.21*e*^−4^

**Table 3:** Key acupoint set *U* output by each algorithm and corresponding network loss *C*(*U*).

	Key_node	Degree	CC	BC	EC	k-shell	CLD
1	GV14	GV14	GV14	GV26	GV14	GV14	GV14
2	GV20	GV20	GV20	GV14	GV20	GV20	GV20
3	EX-HN5	EX-HN5	EX-HN5	EX-HN5	EX-HN5	EX-B2	EX-B2
4	EX-B2	EX-B2	EX-B2	EX-B2	EX-B2	EX-UE12	EX-HN5
5	CV4	CV23	CV23	CV23	CV23	CV6	CV4
6	CV6	CV6	CV22	CV12	CV4	CV4	CV23
7	PC6	PC6	PC6	PC6	PC6	PC6	PC6
8	PC5	PC5	PC5	PC5	PC5	PC5	PC5
9	TE5	TE5	TE5	TE5	TE5	TE17	TE5
10	TE17	TE17	TE17	TE17	TE17	TE5	TE17
11	HT7	HT7	HT7	HT7	HT7	HT7	HT7
12	HT5	HT5	HT5	HT5	HT5	HT5	HT5
13	SI3	SI3	SI3	SI3	SI3	SI3	SI3
14	SI11	SI11	SI1	SI11	SI11	SI9	SI9
15	LU11	LU11	LU11	LU11	LU11	LU11	LU11
16	LU5	LU7	LU7	LU7	LU7	LU5	LU10
17	LI4	LI4	LI4	LI4	LI4	LI4	LI4
18	LI11	LI11	LI11	LI11	LI11	LI11	LI11
19	LR3	LR3	LR3	LR3	LR3	LR3	LR3
20	LR2	LR14	LR2	LR14	LR2	LR2	LR2
21	GB20	GB20	GB20	GB20	GB20	GB34	GB34
22	GB34	GB34	GB34	GB34	GB34	GB30	GB31
23	KI3	KI3	KI3	KI3	KI3	KI3	KI3
24	KI6	KI6	KI6	KI6	KI6	KI6	KI6
25	BL23	BL23	BL23	BL23	BL23	BL23	BL23
26	BL32	BL32	BL25	BL57	BL13	BL20	BL32
27	SP6	SP6	SP6	SP6	SP6	SP6	SP6
28	SP9	SP9	SP9	SP9	SP9	SP9	SP9
29	ST36	ST36	ST36	ST36	ST36	ST36	ST36
30	ST25	ST25	ST25	ST25	ST40	ST6	ST4

*C*(*U*)	5.347*e*^−1^	5.417*e*^−1^	5.353*e*^−1^	5.305*e*^−1^	5.303*e*^−1^	5.254*e*^−1^	5.281*e*^−1^

**Table 4:** The top five most frequent diagnoses and prescribed acupoints.

Diagnosis pattern code frequency (*n*)	Acupoint frequency (%)				
U61 460	ST36 32 (7.0%)	ST35 27 (5.9%)	LI4 26 (5.7%)	SP6 25 (5.4%)	SP10 21 (4.6%)
U62 432	LI4 38 (8.8%)	ST36 37 (8.6%)	LR3 36 (8.3%)	KI3 25 (5.8%)	SP6 21 (4.9%)
U71 424	KI3 40 (9.4%)	SP6 25 (5.9%)	KI7 18 (4.2%)	BL23 16 (3.8%)	LI4 15 (3.5%)
U65 397	LR3 49 (12.3%)	LI4 38 (9.6%)	CV17 26 (6.5%)	GV20 24 (6.0%)	SP6 24 (6.0%)
U63 378	ST36 39 (10.3%)	LI4 33 (8.7%)	CV12 32 (8.5%)	PC6 24 (6.3%)	ST40 21 (5.6%)

## Data Availability

The data used to support the findings of this study are included within the article.  Section 2.1 describes the data sources of this study in detail. The data supporting this manuscript have been obtained from previously reported studies and datasets, which have been cited. The data are also available from the following weblink: kns.cnki.net/kcms/detail/detail.aspx?FileName=WXZZ403.003&DbName=CJFQ1994. Furthermore, the processed data are available from the corresponding author upon request.
